# Quercetin Increases MUC2 and MUC5AC Gene Expression and Secretion in Intestinal Goblet Cell-Like LS174T via PLC/PKCα/ERK1-2 Pathway

**DOI:** 10.3389/fphys.2018.00357

**Published:** 2018-04-06

**Authors:** Simona Damiano, Anna Sasso, Bruna De Felice, Ilaria Di Gregorio, Giuliana La Rosa, Gelsi A. Lupoli, Anna Belfiore, Paolo Mondola, Mariarosaria Santillo

**Affiliations:** ^1^Dipartimento di Medicina Clinica e Chirurgia, Università di Napoli “Federico II”, Naples, Italy; ^2^Dipartimento di Scienze e Tecnologie Ambientali Biologiche e Farmaceutiche, Università della Campania Luigi Vanvitelli, Caserta, Italy

**Keywords:** quercetin, MUC2, MUC5AC, goblet cells, mucin secretion, gastrointestinal barrier

## Abstract

The main dietary flavonoid quercetin, is known to preserve the integrity of gastrointestinal barrier and to have anti-inflammatory, anti-cancer, anti-fibrotic, and other beneficial properties. Many of the biological effects of quercetin appear to be associated to the modulation of cell signaling pathways, rather than to its antioxidant activity. In spite of the large number of data available on the molecular and cellular mechanisms by which quercetin exerts its biological effects, including protection of intestinal barrier function, there is a lack of data about the role of this substance on the expression and/or the secretion of mucins released by intestinal goblet cells. Here we investigated the effects of quercetin on the secretion and the gene expression of the main intestinal gel-forming mucins, MUC2 and MUC5AC, and the signaling mechanisms underlined, in human intestinal goblet cell-like LS174T. We found that quercetin increases intracellular Ca^2+^ levels and induces MUC2 and MUC5AC secretion in a Ca^2+^-dependent manner. Quercetin also induces mRNA levels of both secretory mucins. Quercetin stimulation of LS174T cells increases phosphorylation levels of extracellular signal regulated kinase (ERK)1-2 and protein kinase C (PKC) α and the induction of MUC2 and MUC5AC secretion and mRNA relies on phospholipase C (PLC), PKC, and ERK1-2 signaling pathways since the PLC inhibitor U73122, the PKC inhibitor bisindolylmaleimide (BIM) and the ERK1-2 pathway inhibitor PD98059, all revert the stimulatory effects of quercetin. We also demonstrated that the induction of mucin gene expression by quercetin is not limited to goblet cells. Indeed, quercetin induces mRNA levels of MUC2 and MUC5AC via PKCα/ERK1-2 pathway also in the human intestinal epithelial Caco-2 cells. These data highlight a novel mechanism thereby quercetin, regulating the secretory function of intestinal goblet cells and mucin levels in enterocytes may exert its protective effects on intestinal mucosal barrier.

## Introduction

Intestinal mucosal barrier function represents the ability of the intestine to provide the absorption of nutrients and other substances while preventing bacterial invasion and the damaging effects of toxic molecules that transit through the gastrointestinal tract. Alteration of intestinal barrier function and increased intestinal permeability is associated to a variety of immune-mediated diseases like inflammatory bowel disease, comprising ulcerative colitis, and Crohn's disease (Kaser et al., 2010). The main targets of molecules controlling mucosal homeostasis are the complex protein-protein networks that form cell junctions, mainly tight junctions (Turner, 2009) and the mucins, the most important constituents of mucus layers. Secretory and membrane-bound mucins are large glycosylated proteins important in the mainteinance of the intestinal epithelial homeostasis (Johansson and Hansson, 2016). The main secretory mucins, MUC2 and MUC5AC lack of a trans-membrane domain and are secreted in the extracellular space (Singh and Hollingsworth, 2006). This group of mucins form large polimers with gel-like properties that confer to the mucous layer protective and lubricant properties. Mucin expression is tissue and cell type specific. Both MUC2 and MUC5AC are expressed and secreted by goblet cells (Johansson et al., 2013). MUC2, is the major intestinal gel-forming mucin of the loose unattached mucus layer of small intestine. Before its calcium-dependent release in the intestinal lumen MUC2 is stored in the goblet cell granulae (Ambort et al., 2012). In this tract, mucus and liquid secretion associated to motor activity limits bacterial exposure of the epithelium that by this way is kept sterile. MUC2 is also expressed in both the outer unattached or the inner adherent mucus layers of colon. MUC5AC, is mainly present at the inner mucous layer of gastric mucosa, but it is also expressed at some extent in the small intestine and colon (Van Klinken et al., 1995).

Mucus secretion is highly modulated by luminal substances including bioactive compounds like polyphenols present in functional foods and by intestinal microbiota. We focused our attention on the effects of quercetin (3, 3′, 4′, 5, 7-pentahydroxyflavone), a low molecular weight hydrophobic flavonoid mainly presents in fruit and vegetables, on mucin expression and secretion by human intestinal goblet cells.

Quercetin has antioxidant properties, but recent studies designed to deepen the molecular mechanisms that underlie its biological effects have demonstrated that they appear to be related to the ability of quercetin to modulate cell-signaling pathways, rather than to its antioxidant activity.

Quercetin also enhances barrier integrity in Caco-2 cells inducing remodeling of epithelial tight junctions (Valenzano et al., 2015). Quercetin, is also able to protect the gastric mucosa against a variety of ulcerogenic agents. Yan et al. (2011) reported a protective effect of a quercetin glycoside (quercetin 3-O-β-D-glucuronopyranoside) on indomethacin-induced gastric mucosal injury in the rat, through the induction of mucus secretion. However, nowadays, no data are available about the effects of this flavonoid on the secretory function of intestinal goblet cells.

In this study we investigated the effects of quercetin on gene expression and secretion of the main intestinal secretory mucins MUC2 and MUC5AC, in human intestinal goblet cell-like LS174T cells and the signaling pathways underlined. To evaluate whether quercetin effects are not limited to goblet cells, we also evaluated the modulation of mucin gene expression by quercetin in intestinal epithelial Caco-2 cells.

## Materials and methods

### Material

Quercetin (chemical purity ≥ 95%, HPLC), Alcian Blue, Carbamoylcholine chloride (Cch) and fluorescent probe DPBA (2-Aminoethyl diphenylborinate) were purchased from Sigma-Aldrich (USA). Periodic acid-Schiff (PAS) was purchased from Dako-Products, BAPTA-AM, U73122, bisindolylmaleimide (BIM) and PD98059 were purchased from Calbiochem (USA).

### Cells cultures

LS174T and Caco-2 cells were obtained from the ATCC (American Type Culture Collection, USA) and routinely grown in 75 cm2 flask. LS174T were grown in Advanced modified Eagle medium (A-MEM, GIBCO) containing 4.5 g/l glucose, supplemented with 10% fetal bovine serum, 2 mM L-glutamine, 100 U/ml penicillin and 100 g/ml streptomycin (Sigma-Aldrich). Caco-2 cells were grown in Dulbecco's modified Eagle medium (DMEM, Sigma-Aldrich, USA), containing 4.5 g/l glucose, supplemented with 10% fetal bovine serum, 1% nonessential amino acids, 2 mM L-glutamine, 100 U/ml penicillin, and 100 g/ml streptomycin (Sigma-Aldrich, USA). The cells were kept in a 5% CO_2_ and 95% air atmosphere at 37°C.

### Western blotting analysis

LS174T and Caco-2 cells were grown to semi-confluence in 60-mm dishes in presence or absence of quercetin (25/50 μM), then were washed twice with PBS and cell lysates were obtained in RIPA buffer, containing 50 mM Tris–HCl pH 7.5, 150 mM NaCl, 1% NP40, 0.5% deoxycholate, 0.1% SDS, 2.5 mM Na-pyrophosphate, 1 mM β-glycerophosphate, 1 mM NaVO4, 1 mM NaF, 0.5 mM Phenylmethylsulfonyl fluoride (PMSF) and a cocktail of protease inhibitors (Roche, USA). The cells were kept for 15 min at 4°C and disrupted by repeated aspiration through a 21-gauge needle. Cell lysates were centrifuged for 15 min at 13,000 rpm and the pellets were discarded. The protein concentration was determined by Lowry assay. Fifty micrograms of total proteins were subjected to sodium dodecyl sulfate 7.5% polyacrylamide gel electrophoresis (SDS-PAGE) under reducing conditions. Filters were incubated with specific rabbit polyclonal antibody that cross reacts with p-PKCα (Ser657, Upstate) or a specific rabbit polyclonal antibody against p-Erk1-2 (Santa Cruz Biotecnology, INC.), then incubated with a peroxidase-conjugated anti-rabbit or anti-mouse secondary antibody (GE-Healthcare, UK). Peroxidase activity was detected with the enhanced chemiluminescence (ECL) system (GE-Healthcare). Protein bands were revealed by ECL and, when specified, quantified by densitometry using Scion Image software. Densitometric values were normalized to α-tubulin.

### Slot-blot analysis

The supernatants of LS174T were loaded onto the BIO-Dot SF apparatus according to the protocol provided by the manufacturer (BIO-RAD). Filters were incubated with specific mouse polyclonal rabbit antibodies that cross reacts with MUC2 or MUC5AC (Abcam), before being washed three times in Tris-Buffered Saline-Tween 20 0.1% (TBS-T) and then incubated with a peroxidase-conjugated secondary rabbit antibody (GE-Healthcare, UK). After washing with TBS-T peroxidase activity was detected with the enhanced chemiluminescence (ECL) system (GE-Healthcare). Protein bands revealed by ECL were quantified by densitometry, using Scion Image software; densitometric values were normalized for intracellular protein content, that was unchanged in samples treated with quercetin respect to controls. Moreover, total protein content of the cell media from quercetin treated cells were comparable to that of control samples.

### Intracellular calcium flux assay

Intracellular calcium levels in LS174T cells were measured using the Fluo-4 NW calcium indicator kit (Life Technologies), according to the manufacturer's instructions. Briefly, the LS174T cells were grown in 96 well microplates (20000/w) for 18 h, then were washed with assay buffer and loaded with Fluo-4 probe in the presence of 5 mM Probenecid for 30 min at 37°C and then 30 min at room temperature. Fluorescence was recorded every 6 s using the Flouroskan Ascent–FL (Thermo electronic corporation) with excitation at 485 nm and emission at 538 nm. Fluorescence was recorded for 1 min before loading cells with quercetin or carbachol and was then monitored for an additional 10 min. Blank samples fluorescence was also measured and subtracted from all experimental points.

### RNA extraction from LS174T and Caco-2 cell line

Ten micrograms of total RNA from LS174T and Caco-2 cells were obtained. Trizol (Invitrogen, no. 15596-026) method has been used for isolation and purification of RNA. RNA was isolated including a DNase digestion step. These standardized RNA isolation procedures guarantee high-quality RNA. Using the Agilent 2100 Bioanalyzer platform (Agilent Technologies) RNA samples were quality-checked documenting the identification of 18-S and 28-S ribosomal RNA (rRNA) peaks. The yields were 9–15 μg, and the RNA Integrity Number (RIN) was between 8.2 and 10.

### Quantitative reverse transcription-PCR (real-time RT-PCR)

To confirm the expression patterns of genes MUC2 and MUC5AC, we performed a quantitative RT-PCR using the comparative Ct method. Transcript levels of the target genes were normalized to G6PD (the internal control) after correcting for differences in amplification efficiencies. RT-PCR reactions (*n* = 3) were performed for each gene of interest using a 7500 Fast Real-Time PCR system (Applied Biosystems, Foster City, CA, USA). All genes investigated have previously been identified and sequences were available in GenBank. Primers for qRT-PCR analysis were designed using the Primer3 program (http://www.bioinformatics.nl/cgi-bin/primer3plus/primer3plus.cgi).

The final PCR reactions contained: 0.4 μM of each primer; 0.25 × SYBR Green (Invitrogen); 4 mM MgCl_2_ and as template 5 μl of cDNA reverse transcribed from a standardized amount of total RNA (0.3 μg). qRT-PCR was performed using Hotstart Taq polymerase (Qiagen) in a final volume of 20 μl. All quantitative reactions were subjected to: 95°C for 15 min followed by 45 cycles at 94°C for 15 s, 59°C 15 s and 72°C 15 s. Melting curve analysis was applied to all reactions to ensure homogeneity of the reaction product.

Potential contamination was assessed by including non-reverse transcribed total RNA (genomic DNA contamination) and controls without template, observing no products in these reactions.

Primers used in these studies are the following: Human MUC2: (F), CGA CTA CTA CAA CCC TCC GC (R), GGG AGGAGT TGG TAC ACA CG; Human MUC5AC: (F), GAC TGT CAT CCT CTG TGC G (R), CAC CTCGTA GTT GAG GCA CA; Human G6PD: (F), ACA GAG TGA GCCC TTC TTC AA (R), ATA GGAGTT GCG GGC AAA G.

### Flow cytometric analysis of flavonoids using DPBA (2-aminoethyl diphenylborinate) probe

Cells were grown to semiconfluency in 60-mm culture dishes. Following the method of Grootaert et al. (2016), after detachment by trypsin, cells were suspended in 1 mL of phosphate buffered saline (PBS) and fixed overnight with 1% formaldehyde at room temperature. Next, cells were incubated with the fluorescent probe DPBA, (0.2%) in DMSO (0.3%), for 1 h at room temperature, whereas control cells were incubated whit DMSO (0.3%) in water without stain. After two washes in PBS, the samples were resuspended in 500 μl of PBS and analyzed by flow cytometry using FACSCAN (BD, Heidelberg, Germany) and data were analyzed using Flowing 2.5.1 software.

### Periodic acid-Schiff (PAS) cytochemical staining for glycoproteins

LS174T cells were grown for 24 h on glass coverslip. Then, the medium was removed and cells immediately fixed in 3.7% Paraformaldehyde. Next, the cells were oxidized with 1% periodic acid (Sigma-Aldrich, USA) for 10 min. After washing with water, cells were treated with Schiff's reagent (Dako-Products) for 20 min and then rinsed with water. The cells were counterstained with hematoxylin (Sigma-Aldrich, USA) and then the coverslips were briefly washed with distilled water, and finally mounted on glass slides for microscopy examination. Cells were analyzed with an optical microscope (20x).

### Alcian blue at pH 2.5 cytochemical staining for glycosaminoglycans (GAGs)

LS174T cells were grown for 24 h on glass coverslip. The day after the medium was removed and cells fixed in 3.7% Paraformaldehyde. Then the cells were incubated in Acetic Acid Solution (3%) for 3 min and next with Alcian Blue (pH 2.5) solution for 30 min at room temperature. Following the treatment, the cells were washed in Acetic acid solution to remove Alcian Blue excess and then in water. The coverslips were mounted on glass slides for microscopy examination. Cells were analyzed with an optical microscope (20x).

### Periodic acid-Schiff's staining for glycoprotein bands

The supernatants of LS174Tcells, grown in absence and in presence of quercetin (50 μM), were loaded onto the BIO-Dot SF as previously described. Next the membranes were placed in 5% non-fat milk in tris buffered saline, 0.1% Tween 20 (TBST, Bio-Rad Laboratories) at room temperature overnight to block the non-specific binding sites. Filters were incubated in 1% periodic acid for 15 min, rinsed with distilled water and stained with Schiff's reagent for up to 60 min at room temperature. Glycoprotein bands were quantified by densitometry using ImageJ software.

### Alcian blue at pH 2.5 staining for glycosaminoglycan bands

Slot-Blot membranes of LS174T supernatants cells, obtained and treated as previously described, were incubated in Acetic Acid Solution (3%) for 3 min. Then membranes were washed with distilled water and stained with Alcian Blue (pH 2.5) solution for 30 min at room temperature. Glycosaminoglycan bands were quantified by densitometry using ImageJ software.

### Statistical analysis

Statistical differences were evaluated using a Student's *t*-test for unpaired samples.

## Results

### Quercetin increases MUC2 and MUC5AC secretion and mRNA levels

**Figure 1:**
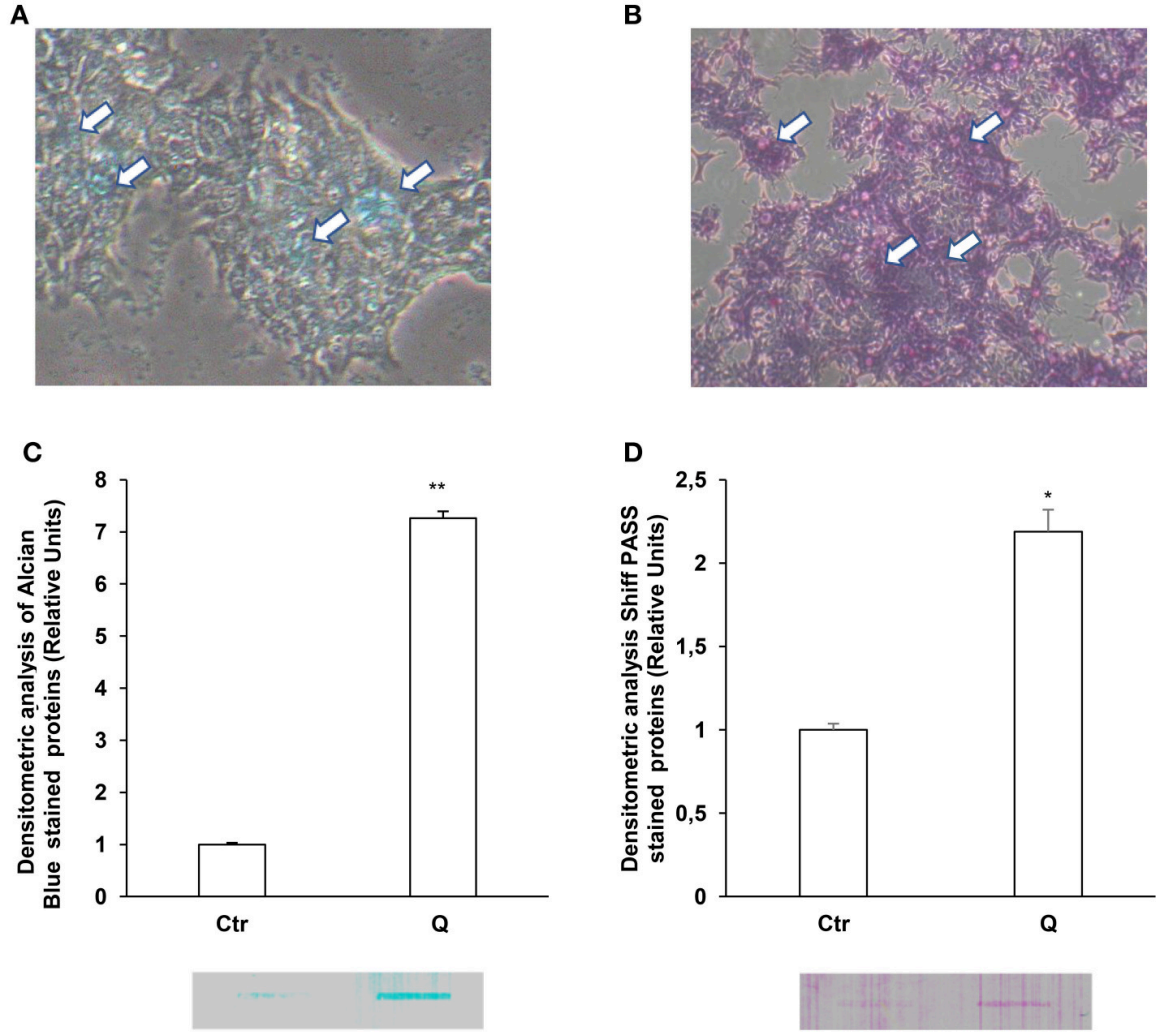
Quercetin induces glycoprotein and glycosaminoglycan secretion in LS 174T. Periodic acid-Schiff (PAS) **(A)** and Alcian blue **(B)** staining of LS 174T cells. Arrows indicate intracellular mucous granules. **(C,D)** Slot Blot analysis of glycoproteins and GAGs in LS174T cell medium. Cells were incubated with MEM medium without FBS for 24 h and stimulated with 50 μM quercetin and membranes were stained with Periodic acid-Schiff (PAS) **(C)** and Alcian blue **(D)**. The histogram shows the mean ± SE (*n* = 3) values of GAGs **(C)** and glycoproteins **(D)** levels obtained by densitometric analysis of protein bands and normalized to the respective intracellular protein concentrations of three independent experiments. ^*^p < 0.05 vs. Ctr, ^**^p < 0.001 vs. Ctr.

**Figure 2:**
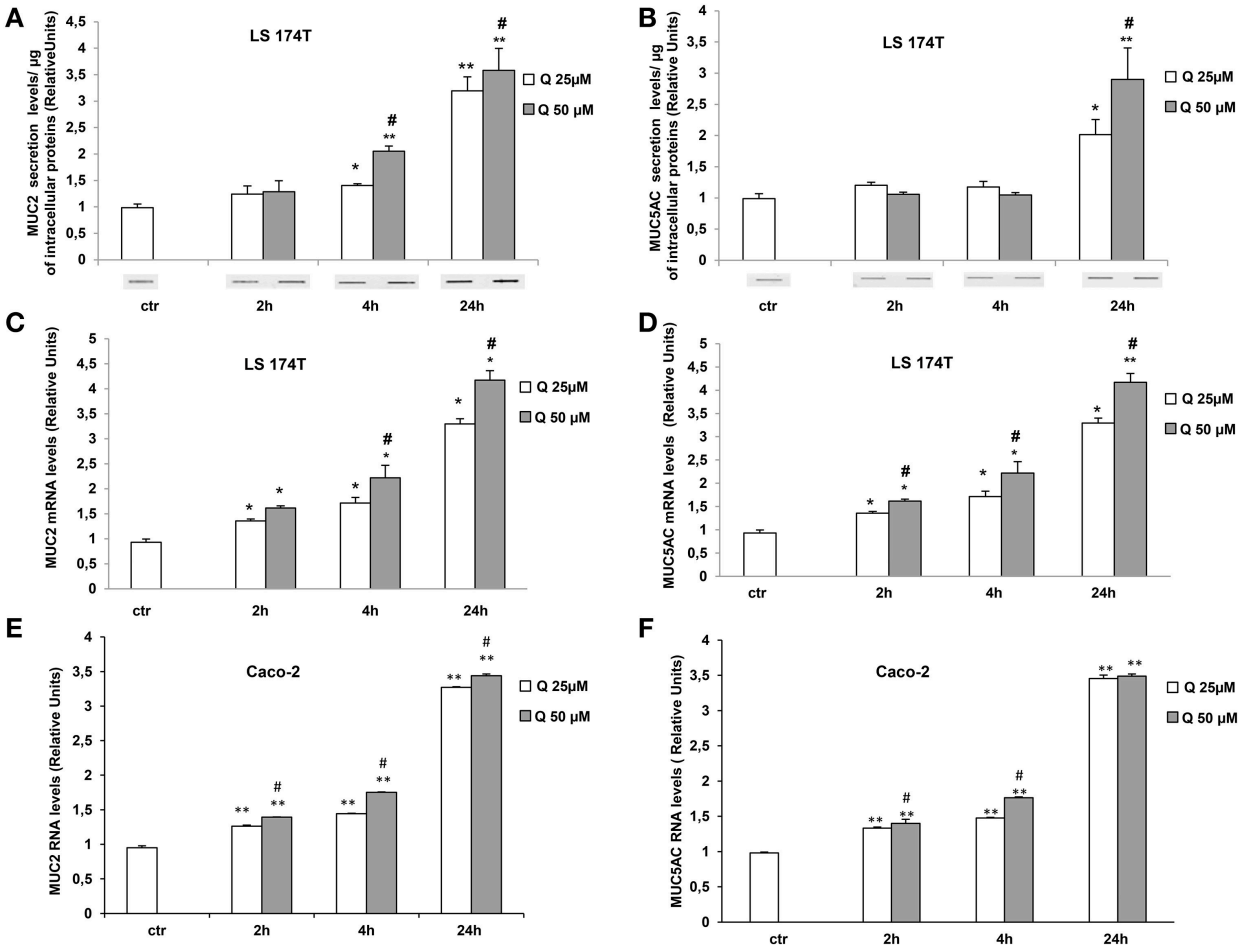
Quercetin increases MUC2 and MUC5AC secretion and mRNA levels. **(A,B)** Slot-Blot analysis of LS174T cell medium. Cells were incubated with MEM medium without FBS for 24 h and stimulated with 25 (Q25) or 50 (Q50) μM quercetin at the indicated times. The histogram shows the mean ± SE (*n* = 3) values of MUC2 **(A)** and MUC5AC **(B)** levels obtained by densitometric analysis of protein bands and normalized to the respective intracellular protein concentrations of three independent experiments. ^*^p < 0.05 and ^**^p < 0.001 vs. Ctr, #*p* < 0.05 vs. 25 μM of the corresponding time point. Real-time-PCR analysis of MUC2 and MUC5AC m-RNA levels in LS174T **(C,D)** and in Caco-2 **(E,F)** cells treated at the times indicated with 25 and 50 μM quercetin in medium without serum. Expression values were normalized using glucose-6-phosphate-dehydrogenase mRNA(G6PD) The histogram shows the mean ± SE (*n* = 3) of RT-PCR of three independent experiments. ^*^p < 0.05 and ^**^p < 0.001 vs. Ctr, #*p* < 0.05 vs. 25 μM of the corresponding time point.

Since LS174T cells produce large amount of secretory mucins released in the extracellular space (Van Klinken et al., 1996) we used these cells to study the effects of quercetin on mucin secretion. These cells are similar to intestinal goblet cells since they contain intracellular mucous granules as shown by Alcian blue and Shiff-PAS staining for glycoproteins and glycosaminoglycans, respectively (Figures 1A,B). By Slot-Blot of cell media and staining of the membranes with Alcian blue and Shiff-PAS, we also observed that in LS174T cells the treatment with 50 uM quercetin for 24 h increases glycoproteins and glycosaminoglycans secretion (Figures 1C,D). To evaluate the effects of quercetin on MUC2 and MUC5AC secretion, LS174T cells were incubated for 2, 4, or 24 h with two different concentrations of quercetin, 25 and 50 μM, and then mucin secretion was measured by Slot Blot analysis. Secretion levels of MUC2 protein after 4 and 24 h incubation with both doses of quercetin were significantly higher than that of untreated control cells (Figure 2A). MUC5AC secretion levels were significantly increased in cells incubated with 25 and 50 μM of quercetin for 24 h (Figure 2B). compared to control cells. In these experimental conditions quercetin did not affect cell viability as shown by trypan blue staining of cells (Supplementary Figure 1A), indicating that the increased levels of secreted mucins cannot be ascribed to cell lysis.

We also evaluated the effect of quercetin on MUC2 and MUC5AC gene expression (Figures 2C,D). The mRNA levels of MUC2 and MUC5AC measured by RT PCR were significantly increased by the treatment with 25 and 50 μM quercetin respect to untreated control samples and the increase was time-dependent. Further studies were carried out in intestinal epithelial Caco-2 cells. Similar induction of MUC2 and MUC5AC mRNA levels were observed in Caco-2 cells treated with quercetin (Figures 2E,F). Even in these cells, quercetin did not affect cell viability (Supplementary Figure 1B).

**Figure 3:**
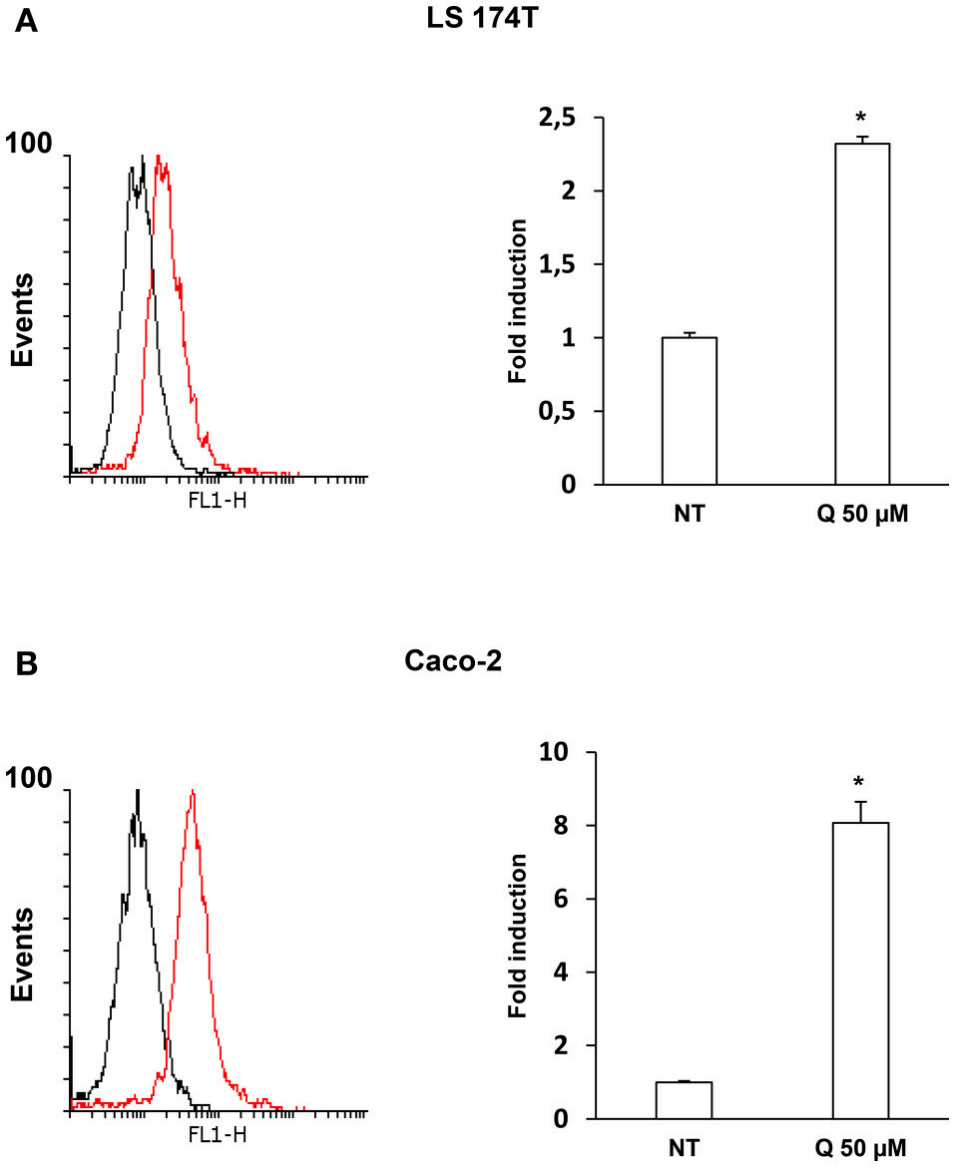
Quercetin permeates LS 174T **(A)** and Caco-2 **(B)** cells membrane. Flow cytometric analysis using fluorescent probe DPBA for the staining of flavonoids, 10,000 cells were counted for each sample. The graph shows the mean ± SE (*n* = 3) values of three independent experiments. The insert shows the histograms of a representative experiment; the red histogram denotes the sample treated with 50 μM of Quercetin. ^*^p < 0.001 vs. Ctr.

To evaluate whether quercetin permeates LS174T and Caco-2 cell membranes flow cytometry experiments using the fluorescent probe DPBA for the staining of flavonoids (Grootaert et al., 2016) were performed. The Figures 3A,B shows a significant increase of DPBA fluorescence in LS174T and Caco2 cell lines incubated with quercetin.

### The induction of MUC2 and MUC5AC secretion by quercetin is Ca^2+^- dependent

**Figure 4:**
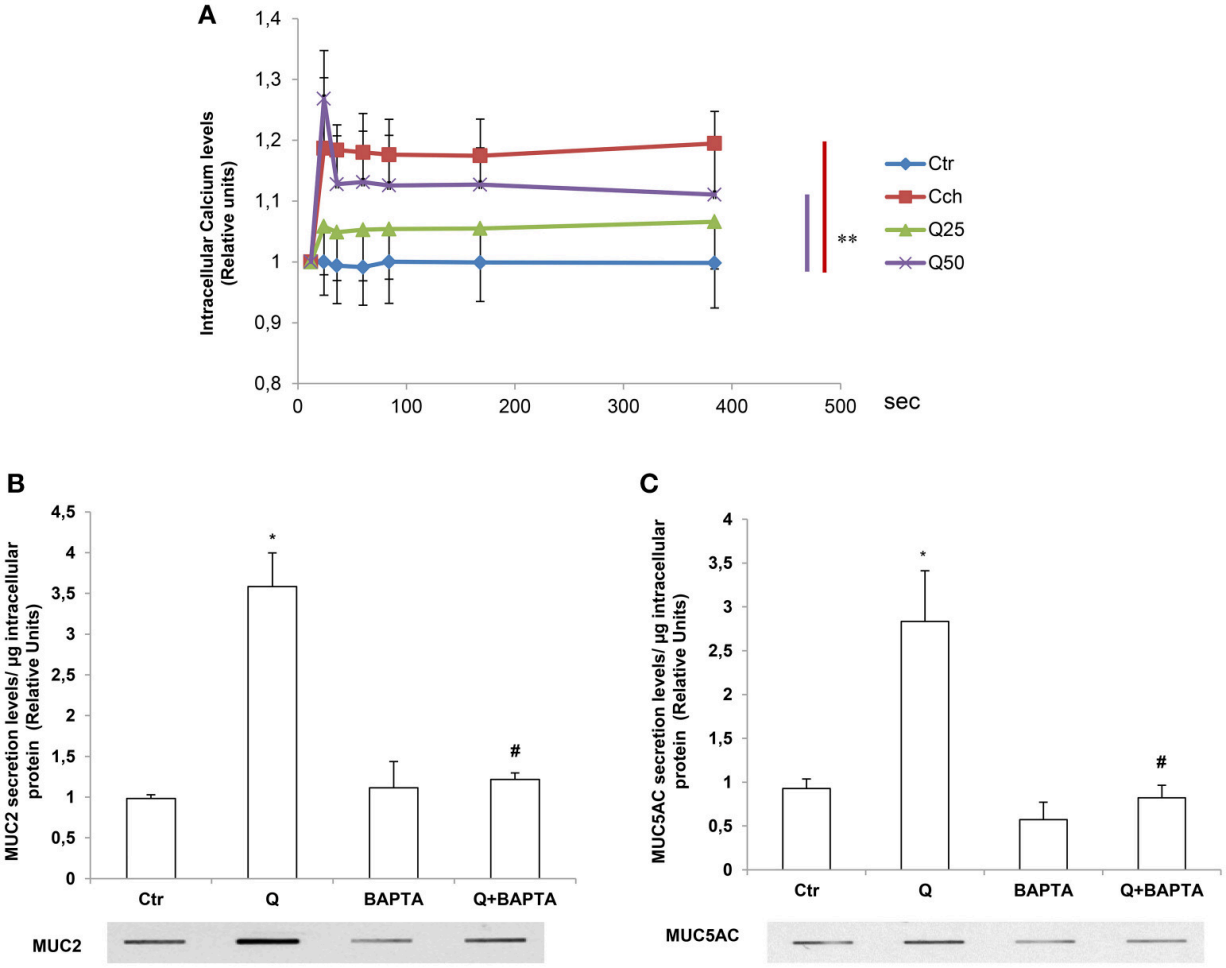
The induction of MUC2 and MUC5AC secretion by quercetin is Ca^2+^- dependent. **(A)** Fluorimetric analysis of intracellular Ca^2+^ levels in LS174T cells stimulated with 25 (25Q) and 50 (50Q) μM quercetin or 1 mM carbachol (Cch). Each point represents the mean ± SE (*n* = 3) of three independent experimental sessions. ^**^p < 0.01 vs. Ctr. **(B,C)** Slot-Blot analysis of incubation medium of LS174T cells grown for 24 h in MEM without serum and stimulated with 50 μM quercetin in the absence or presence of the Ca^2+^ chelator BAPTA-AM. The histogram shows the mean ± SE (*n* = 3) of the MUC2 **(B)** and MUC5AC **(C)** proteins, obtained by densitometric analysis of three independent experiments and normalized for the respective cell protein concentrations. ^*^p < 0.05 vs. Ctr, #*p* < 0.05 vs. Q.

The release of secretory mucins in the extracellular space is a Ca^2+^-dependent event (Yang et al., 2013). For this reason, we evaluated the effects of quercetin on intracellular Ca^2+^ levels by fluorimetry using a FLUO4 fluorescent probe in the presence of Probenecid (5 mM) which has the function of holding the probe inside the cells. The Figure 4A shows intracellular calcium transients kinetics after quercetin stimulation of LS174T cells. In these experiments, 1 mM Cch was used as a positive control to induce a significant and rapid increase of intracellular calcium concentrations. The incubation with 50 μM quercetin induces intracellular Ca^2+^ levels in LS174T cells over time compared to untreated control cells and cells treated with 25 μM quercetin. To demonstrate that quercetin-induced mucin secretion is dependent on the increase of intracellular Ca^2+^ levels, LS174T cells were incubated with 50 μM quercetin in the presence and absence of an intracellular calcium chelator, 1,2-Bis(2-aminophenoxy) ethane-N,N,N′,N′-tetraacetic acid tetrakis (acetoxymethyl ester) (BAPTA-AM), and the respective culture media were analyzed using Slot-Blot analysis (Figures 4B,C). In the presence of BAPTA-AM, quercetin failed to induce the secretion of MUC2 and MUC5AC.

### Phospholipase C (PLC) cascade is required for quercetin-dependent increase of MUC2 and MUC5AC secretion and mRNA levels

**Figure 5:**
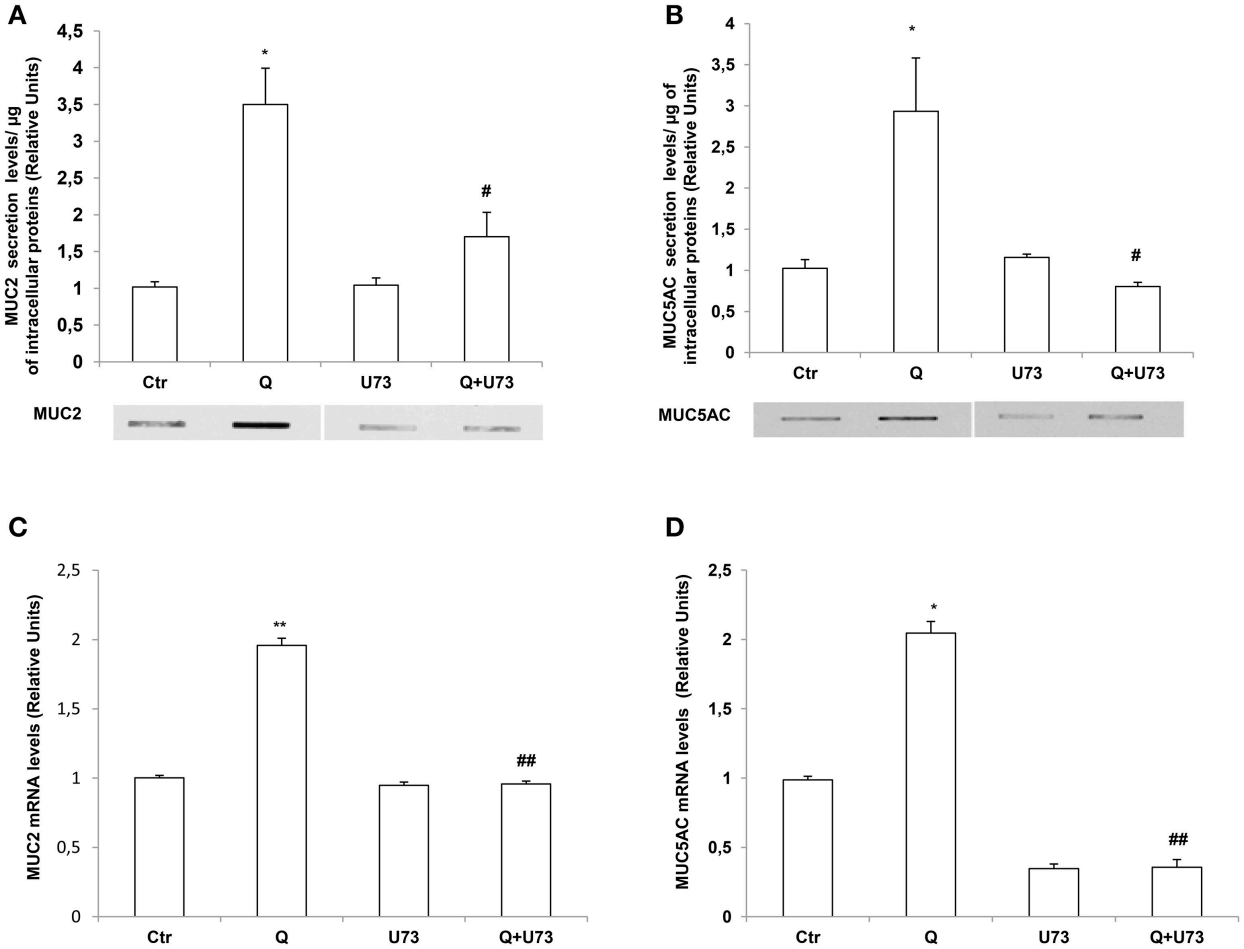
Quercetin induces MUC2 and MUC5AC secretion and mRNA levels through a PLC-dependent pathway. **(A,B)** Slot-Blot analysis of incubation medium of LS174T cells grown for 24 h, in MEM without FBS stimulated with 50 μM quercetin (Q) in the absence or presence of the PLC inhibitor, U73122. The histogram shows the mean ± SE (*n* = 3) of MUC2 **(A)** and MUC5AC **(B)** secretion protein levels obtained by densitometric analysis of three independent experiments and normalized for the respective cell protein concentrations. **(C,D)** Real-time-PCR analysis of MUC2 **(C)** and MUC5AC **(D)** m-RNA levels in LS174T cells treated for 24 h with 50 μM quercetin in the absence or presence of U73122, in medium without serum. Expression values were normalized using glucose-6-phosphate-dehydrogenase mRNA (G6PD). The histogram shows the mean ± SE (*n* = 3) of mRNA levels of three independent experiments. ^*^p < 0.05 vs. Ctr, ^**^p < 0.01 vs. Ctr, #*p* < 0.05 vs. Q, ##*p* < 0.01 vs. Q.

The increase of intracellular calcium by quercetin suggests the involvement of PLC in the signaling pathway mediating the effects of this flavonoid on MUC2 and MUC5AC secretion and expression. PLC enzyme catalyzes phosphatidylinositol 4,5-bisphosphate (PIP2) conversion to inositol-1,4,5-triphosphate's (IP3) and diacylglycerol (DAG) and IP3 is major contributor to intracellular Ca^2+^ release (Tran et al., 2016). Therefore, to evaluate whether PLC signaling mediates quercetin effects we used the PLC inhibitor U73122. Slot Blot and RT-PCR analysis showed that pretreatment of LS174T cells with U73122 completely reversed the increase of secretion (Figures 5A,B) and mRNA (Figures 5C,D) levels of MUC2 and MUC5AC by quercetin.

### Quercetin induction of mucin secretion and gene expression is PKCα and ERK1-2-dependent

**Figure 6:**
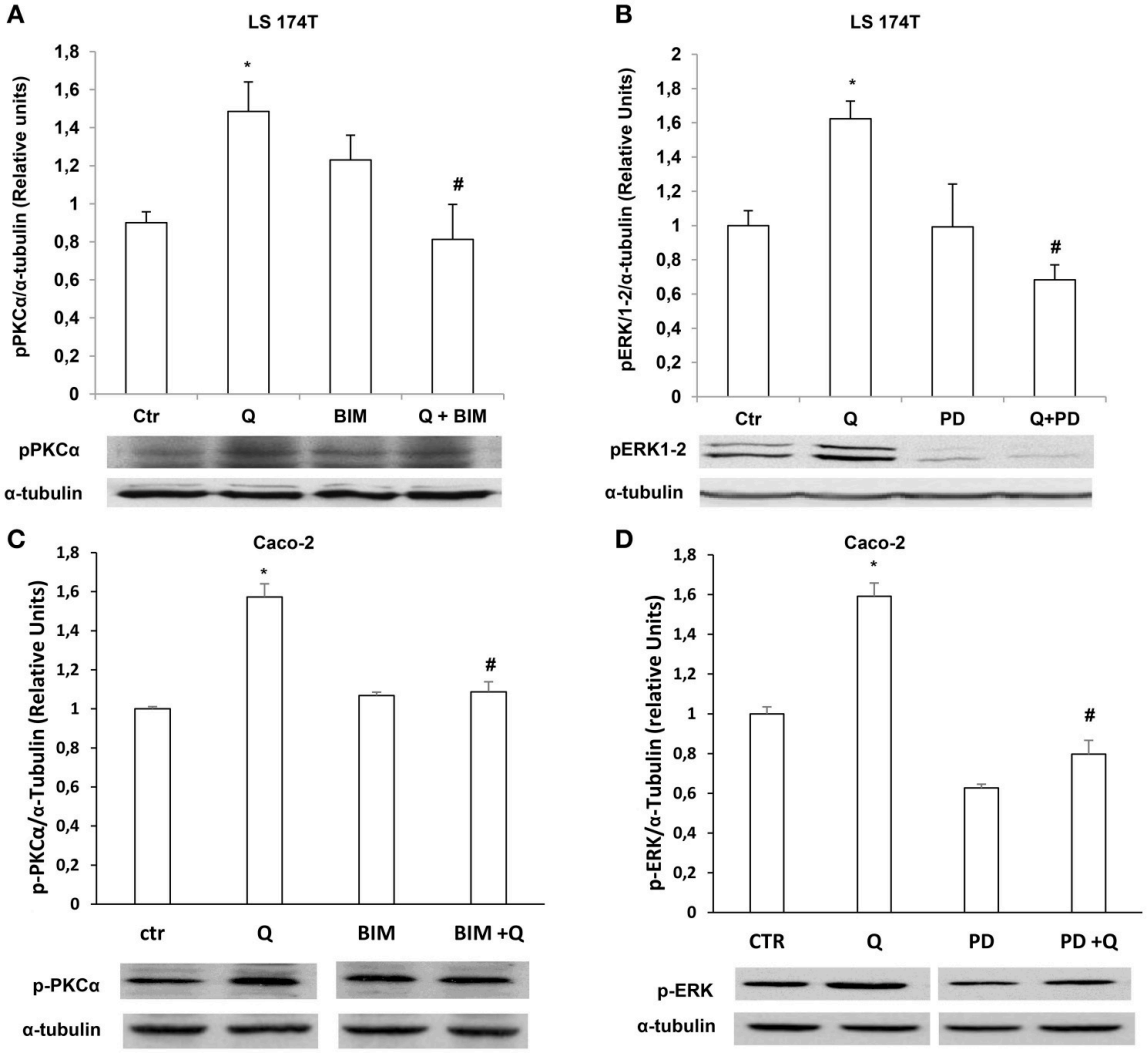
Quercetin activates PKCα and ERK1-2. Western blotting analysis of p-PKCα and p-ERK1-2levels in LS174T **(A,B)** and Caco-2 **(C,D)** cells incubated for 18 h in MEM without FBS, pretreated for 30 min with BIM (PKC inhibitor) or PD98059 (MEK inhibitor) and stimulated with 50 μM of quercetin. The histogram shows the mean ± SE (*n* = 3) obtained by densitometric analysis of p-PKCα and p-ERK1-2 protein bands normalized to α-tubulin of three independent experiments. ^*^p < 0.05 vs. Ctr, #*p* < 0.05 vs. Q.

**Figure 7:**
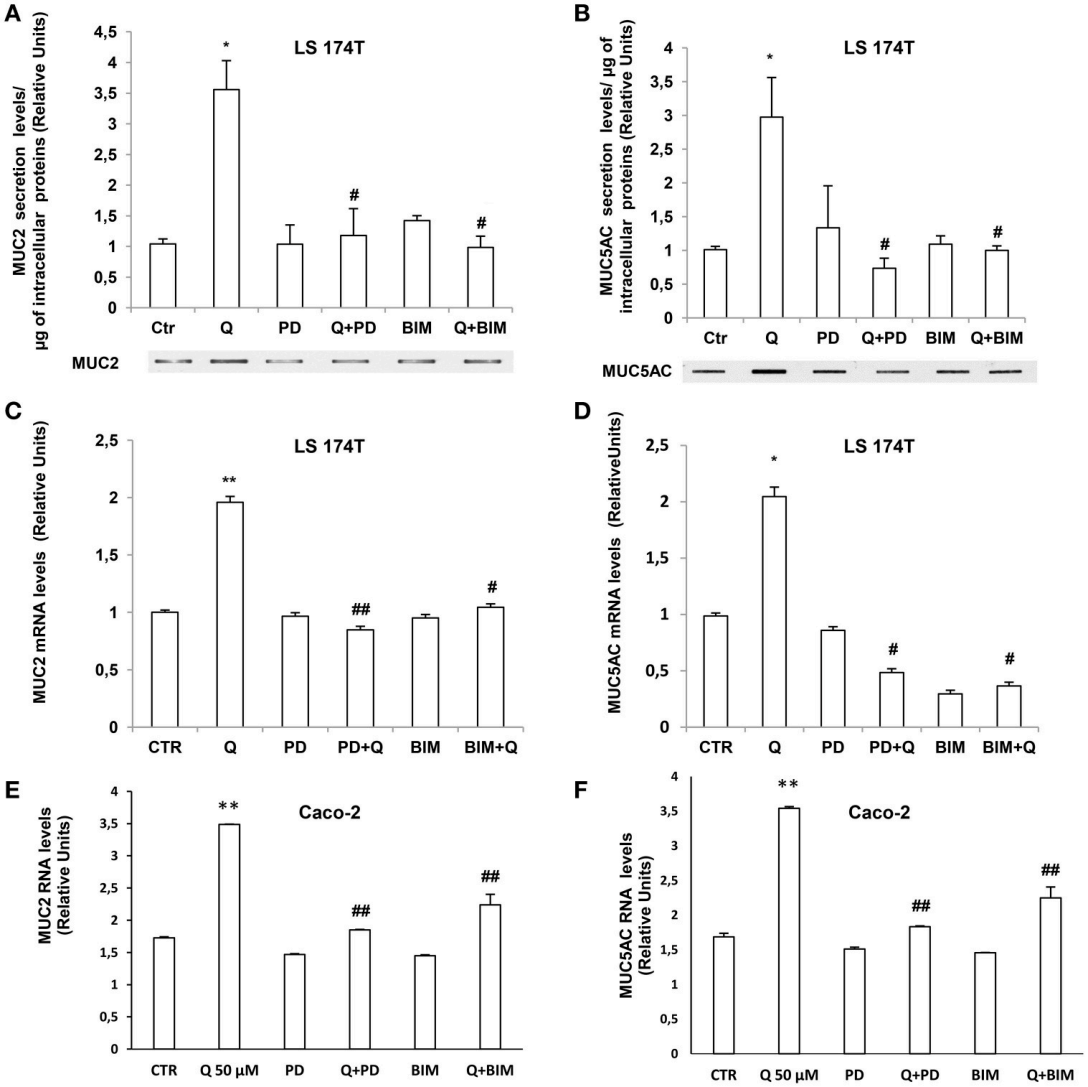
Quercetin induction of mucin secretion and gene expression is PKCα and ERK1-2-dependent. **(A,B)** Slot-Blot analysis of incubation medium of LS174T cells grown for 24 h, in MEM without FBS stimulated with 50 μM quercetin in the absence or presence of BIM (PKC inhibitor) or PD98059 (MEK inhibitor). The histogram shows the mean ± SE of MUC2 **(A)** and MUC5AC **(B)** secretion levels obtained by densitometric analysis of protein bands normalized for the respective cell concentrations of three independent experiments. **(C,F)** Real-time-PCR analysis of MUC2 and MUC5AC m-RNA levels in LS174T **(C,D)** and Caco-2 cells **(E,F)** incubated for 24 h with 50 μM quercetin pretreated with PD98059 or BIM in medium without serum. Expression values were normalized using glucose-6-phosphate- dehydrogenase mRNA (G6PD). The histogram shows the mean ± SE (*n* = 3) of mRNA levels of three independent experiments. ^*^p < 0.05 vs. Ctr, ^**^p < 0.001 vs. Ctr, #*p* < 0.05 vs. Q, ##*p* < 0.001 vs. Q.

We further investigated the molecular mechanisms involved in quercetin-dependent increase of mucin secretion in LS174T and mucin gene expression in LS174T and in Caco-2 cells by evaluating the involvement of ERK1-2 and PKCα signaling pathways. Quercetin significantly induced P-PKCα and P-ERK1-2 levels compared to untreated LS174T (Figures 6A,B) and Caco-2 cells (Figures 6C,D). Furthermore, pretreatment of cells with the PKCα-β-γ inhibitor bisindolylmaleimide (BIM) and the mitogen activated protein kinase kinase (MEK) inhibitor PD98059, prevented the effects of quercetin on the secretion of MUC2 and MUC5AC (Figures 7A,B) in LS174T and mRNA mucin expression (Figures 7A,D), in both cell lines demonstrating that PCKα/ERK1-2 pathway mediates quercetin effects.

## Discussion

Bioactive substances contained in the ingested foods can modulate the epithelial barrier function by preventing permeability loss and cell damage in part through their action on the mucous production. Mucins, the principal component of mucous layer, are important in maintaining intestinal epithelium homeostasis. In the present study, we have demonstrated that quercetin, one of the major dietary flavonoids, increases MUC2 and MUC5AC gene expression and secretion levels in intestinal goblet-like LS174T cells through PLC/PKCα/ERK1-2 pathways; similar effects are exerted by quercetin on MUC2 and MUC5AC gene expression in intestinal epithelial Caco-2 cells. We also found that quercetin increases the levels of secreted glycoproteins and mucopolysaccarydes in LS174T cells (Figure 1). Mucins are highly glycosylated proteins and many evidences support that mucin glycosylation is a key factor promoting mucus barrier integrity and gut microbiota homeostasis (Bergstrom and Xia, 2013). Overall, our data highlight one of the likely mechanism by which this substance exerts its protective effects *in vivo* at the gastrointestinal level.

In plant foods, quercetin is mainly present in the conjugated form as various glycosides (Miles et al., 2014); therefore, the quercetin taken with diet is glycosylated, but in the intestine, it is transformed in the free, highly permeable form. In this study we used the quercetin aglycone which has a much higher membrane permeability than that of the glycosylated one (Perez-Vizcaino et al., 2012). Indeed, flow cytometric quantification by DPBA staining of intracellular flavonoid in LS174T and Caco-2 cells after quercetin incubation, shows that this flavonoid permeates cell membrane (Figures 3A,B). Although we did not highlight the direct molecular targets of quercetin, we can speculate that this flavonoid can exert its biological effects activating intracellular signaling molecules. Accordingly, some literature data report that quercetin cross cell membrane acting on intracellular targets. For example, in mammary estrogen receptor (ER) positive cancer cells, quercetin stimulates cell proliferation through the interaction with the nuclear ERβ (Van der Woude et al., 2005). However, in addition to the binding to ERs, in human breast cancer cell lines quercetin activates signaling pathways through the G-protein-coupled receptor GPR30 (Maggiolini et al., 2004); also, in murine small intestine quercetin activates an opioid receptor (Gim et al., 2015) decreasing amplitudes and frequencies of pacemaker activity of interstitial cells of Cajal. Therefore, it cannot be ruled out that this flavonoid in intestinal cells activates signaling cascade and, in turn, modulates mucin expression and secretion through the interaction with a membrane receptor.

Mucous secretion is dependent upon PLC activation and the resultant increase in the intracellular Ca^2+^ (Abdullah and Davis, 2007; Culp et al., 2011; Liu et al., 2013). In salivary glands muscarinic-induced mucous secretion relies in a first phase on the release of Ca^2+^ from the intracellular stores and in a subsequent phase on the entry of Ca^2+^ from the extracellular space and on PKC. Accordingly, we found that in LS174T cells the secretion of mucins are Ca^2+^ and PLC-dependent (Figures 4,5) as shown by experiments with the calcium chaelator BAPTA and PLC inhibitor U73122. The inhibition of PLC also prevented the induction of MUC2 and MUC5AC mRNA by quercetin.

Literature data report divergent effects of quercetin on ERK1-2. Depending on the cell type, time of stimulation and doses, quercetin inhibits or activates ERK1-2 pathways (Miles et al., 2014). In A549 lung cancer cells, MEK/ERK1-2 activation is required for quercetin induced apoptosis and inhibition of DNA synthesis thus providing a mechanism for the anti-cancer properties of this flavonoid (Nguyen et al., 2004). In LS174T cells the enhanced ERK1-2 activity contributed to the quercetin-dependent increase of MUC2 and MUC5AC protein secretion and mRNA levels, as shown by the observation that the inhibition of ERK1-2 by the MEK inhibitor PD98059 (Figures 6,7) prevented quercetin effects. Similarly, in Caco-2 cells ERK1/2 pathway is involved in quercetin-dependent increase of MUC2 and MUC5AC mRNA levels (Figures 7E,F).

Modulation of reactive oxygen species play a physiological role in redox-sensitive gene expression and cell signaling (Damiano et al., 2013; Accetta et al., 2016; Mondola et al., 2016). Quercetin is a well-known antioxidant, however, at high concentrations it can have pro-oxidant properties (Min and Ebeler, 2009). Many membrane receptors activate redox signaling downstream (Damiano et al., 2012; Viggiano et al., 2012; Terrazzano et al., 2014) Mucin expression is redox sensitive; indeed, we have previously demonstrated that, upon EGF stimulation, reactive oxygen species produced by DUOX enzymes are able to modulate the expression of secretory as well as membrane bound mucins in intestinal human adenocarcinoma Caco-2 cells through the activation of PKCα/ERK1-2 signaling pathways (Damiano et al., 2015). Therefore, the redox properties of this flavonoid could be related to its signaling effects mediating the mucin production and secretion by intestinal cells.

In conclusion, the results of this study carried out *in vitro* in cell cultures, show for the first time the effect of quercetin on intestinal mucins production and the signaling pathways underlined. Although our data need to be further confirmed by *in vivo* experiments in animal models and ultimately in humans, they strongly suggest that dietary supplementation with quercetin, regulating the secretory function of intestinal goblet cells, and mucin gene expression of intestinal epithelial cells, could be a potential therapeutic approach for diseases associated to intestinal barrier disfunction.

## Author contributions

SD and MS: Conceived and designed the experiments; SD, AS, BD, AB, ID, and GLR: Performed the experiments; SD, MS, AS, PM, BD, AB, GL, ID, and GLR: Analyzed the data; GL and BD: Contributed reagents, materials, analysis tools; SD, PM, and MS: Wrote the paper.

### Conflict of interest statement

The authors declare that the research was conducted in the absence of any commercial or financial relationships that could be construed as a potential conflict of interest.

## Abbreviations

BAPTA-AM: 1,2-Bis(2-aminophenoxy)ethane-N,N,N′,N′-tetraacetic acid tetrakis (acetoxymethyl ester)
CCh: Carbamoylcholine chloride
DAG: diacylglycerol
ECL: enhanced chemiluminescence
ERK1-2: extracellular signal regulated kinase
IP3: inositol-1,4,5-triphosphate's
MEK: mitogen activated protein kinase kinase
PIP2: phosphatidylinositol 4,5-bisphosphate
PKC: protein kinase C
PLC: phospholipase C
DPBA: (2-Aminoethyl diphenylborinate).
