# Supplementary material for: Quercetin Increases MUC2 and MUC5AC Gene Expression and Secretion in Intestinal Goblet Cell-Like LS174T via PLC/PKCα/ERK1-2 Pathway
Source: Front Physiol. 2018 Apr 6;9:357. doi: 10.3389/fphys.2018.00357 (PMC5897515; doi:10.3389/fphys.2018.00357)
Supplement: Supplementary file 1 [file Image1.pdf]

Supplementary Material

Quercetin increases MUC2 and MUC5AC expression and secretion in intestinal goblet cell-like LS174T via PLC/PKCα/ERK1-2 pathway

Simona Damiano[1]*, Anna Sasso[1], Bruna De Felice[2], Ilaria Di Gregorio[1], Giuliana La Rosa[1], Gelsi A. Lupoli[1], Anna Belfiore[1], Paolo Mondola[1], Mariarosaria Santillo[1]

*Correspondence: Dr Simona Damiano: simo.damiano@gmail.com

Supplementary material and methods

Cell viability. LS174T and Caco-2 cells viability was evaluated by Trypan blue assay. Briefly, semiconfluent cells incubated at different times (2h, 4h, 24h) in the absence and presence of quercetin (25/50 µM), were suspended in diluted trypan blue (1:1 with PBS) and then immediately counted in Burker's chamber.

Supplementary Figure 1

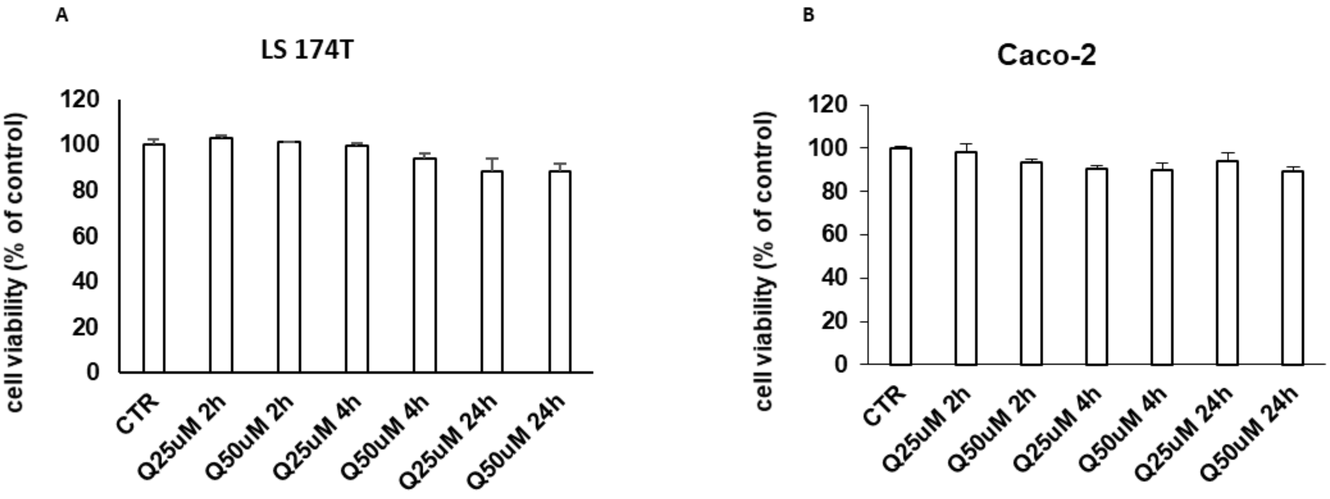

Supplementary Figure 1 Evaluation of cell viability by trypan blue assay in LS174T (**A**) and Caco-2 (**B**) cells. The data are reported as per cent variation compared to control. The data represent the means ± SEM relative to control of three independent experiments.
